# Practical Approaches for Knock-Out Gene Editing in Pigs

**DOI:** 10.3389/fgene.2020.617850

**Published:** 2021-03-05

**Authors:** Laura Daniela Ratner, Gaston Emilio La Motta, Olinda Briski, Daniel Felipe Salamone, Rafael Fernandez-Martin

**Affiliations:** ^1^Laboratorio Biotecnología Animal (LabBA), Departamento de Producción Animal, Facultad de Agronomía, Universidad de Buenos Aires, Buenos Aires, Argentina; ^2^Instituto de Investigaciones en Producción Animal (INPA), CONICET–Universidad de Buenos Aires, Buenos Aires, Argentina

**Keywords:** CRISPR-Cas9, knock-out, electroporation, microinjection, porcine zygotes, SCNT

## Abstract

Pigs are an important resource for meat production and serve as a model for human diseases. Due to their physiological and anatomical similarities to humans, these animals can recapitulate symptoms of human diseases, becoming an effective model for biomedical research. Although, in the past pig have not been widely used partially because of the difficulty in genetic modification; nowadays, with the new revolutionary technology of programmable nucleases, and fundamentally of the CRISPR-Cas9 systems, it is possible for the first time to precisely modify the porcine genome as never before. To this purpose, it is necessary to introduce the system into early stage zygotes or to edit cells followed by somatic cell nuclear transfer. In this review, several strategies for pig knock-out gene editing, using the CRISPR-Cas9 system, will be summarized, as well as genotyping methods and different delivery techniques to introduce these tools into the embryos. Finally, the best approaches to produce homogeneous, biallelic edited animals will be discussed.

## Introduction

In the last few years, there has been a huge impact on porcine biotechnology evolution, evidenced by the numerous pig models developed in this short period of time. Several reviews have been published about gene editing in pigs, from biomedical and agricultural standpoint (Burkard et al., 2018; Yang and Wu, 2018; Lee et al., 2019). In this regard, models to recapitulate human diseases such as arteriosclerosis (Wang et al., 2020), diabetes (Renner et al., 2020) or to test new cancer therapeutics (Kalla et al., 2020) have been developed. Furthermore, gene editing is bringing closer the possibility to use pigs as organ donors for patients on the waiting list for organ transplantation (Lu et al., 2020).

For a long time, the ability to introduce a precise genetic modification in pigs was limited by the available tools. Nowadays, it is possible to induce point mutations in the porcine haplotype, of approximately 2.5 × 10^9^ nucleotides long, through a reverse genetic mechanism. The evolution of genetic modification tools has come a long way. It was initially limited to mice, and later on found a solution in simple bacterial immune mechanisms, the CRISPR-Cas systems. These new molecular tools have been so groundbreaking that have marked the beginning of a new era in genetic manipulation (Doudna and Charpentier, 2014), dividing the history of the generation of modified mammals into “Before and After CRISPR” (BC and AC).

## Evolution of Genetic Modification Tools

The development of genetically engineered animal models was hampered in most species by the lack of appropriate technologies. In BC times, the conventional gene targeting approaches were based on homologous recombination (HR) that are extremely infrequent and whose uses were mostly restricted to mouse model development. With the advent of the CRISPR-Cas system the AC era began, offering novel opportunities to produce genetically engineered animal models. The relevant techniques to enable gene editing in pigs through the years will be discussed in this section.

### Genetic Modification of Animals in BC (Before CRISPR) Times

Several attempts have been made to modify mammalian genomes in the last decades. The first genetically modified mammals were generated by injection of DNA fragments into the male pronucleus (Gordon and Ruddle, 1981; Hammer et al., 1985; and revised in Clark, 2002), where exogenous DNAs were randomly integrated at preexisting double-strand breaks (DSBs), a consequence of the extreme compaction of sperm DNA. Soon after, sperm-mediated gene transfer by *in vitro* fertilization (IVF) was also used to generate genetically modified mammals (Lavitrano et al., 1989); however, this technique could not be replicated by other groups (Brinster et al., 1989). Despite these polemic results, it was later shown that sperm-mediated gene transfer can result in transgenic mammal production when spermatozoa were directly injected into the cytoplasm of the oocyte by intracytoplasmic sperm injection-mediated transgenesis (ICSI-MTG) (Perry et al., 1999; Moreira et al., 2007; Pereyra-Bonnet et al., 2008). However, this technique exhibited limitations related to ICSI species-dependent variable efficiency (reviewed in García-Roselló et al., 2009; Salamone et al., 2017). Later on, it was reported that the cytoplasmic injection of transposon efficiently resulted in transgenic offspring in rodents, pigs, and other large mammals (Sumiyama et al., 2010; Garrels et al., 2011; Furushima et al., 2012; Bevacqua et al., 2017).

Although precise genetic modifications were performed by HR or specific locus integration, their frequencies are usually two or three orders of magnitude lower than a random integration. Thus, the isolation of homologous recombinant cell clones requires long and complex protocols of enrichment, independently of the target locus, based on a combination of positive and negative selections (Thomas and Capecchi, 1987). Nevertheless, these protocols were practically restricted to mouse embryonic stem (ES) cells (Evans and Kaufman, 1981). Cells could be injected into blastocysts generating chimeras with the colonizing the germline (Bradley et al., 1984). Finally, by mating these chimeric animals, it was possible to obtain homogeneous transgenic progeny. The application of this technology in domestic species was limited, because only recently, ES cells from cow were isolated (Bogliotti et al., 2018) and porcine expanded potential stem cells were developed thanks to an exhaustive effort of several groups that tested around 400 combinations of 20 small molecule inhibitors and cytokines (Gao et al., 2019); however, to date no large domestic animals have been obtained with a total or partial contribution of any kind of stem cells yet.

Dolly’s birth (Wilmut et al., 1997) brought the attention to somatic cell nuclear transfer (SCNT) as a new possibility to generate transgenic animal models, such as sheep (Schnieke et al., 1997), cows (Cibelli et al., 1998) and pigs (Onishi et al., 2000), since fetal or adult somatic donor cells can be genetically modified prior to nuclear transfer. Although SCNT could also theoretically allow the generation of knock-out animal models, the complex selection protocols to generate specific integrations resulted in very few gene knock-outs produced by this method in pigs: two monoallelic (Dai et al., 2002; Lai et al., 2002) and two biallelic pigs (Rogers et al., 2008; Prather et al., 2013) have been reported.

Another strategy proposed to induce genetic modifications involves the use of endonucleases that can recognize more than 16 bases and make a single cut per genome (by hazard one cut every 4^16^ bases, approx. every 4 × 10^9^ bases or 1 cut per haploid mammalian genome). The first genome-editing strategy was based on the use of *I-Sce*I, a yeast meganuclease with a recognition site of 18 base pairs (Jacquier and Dujon, 1985). In this regard, Choulika et al. (1995) demonstrated an increase in HR in mammalian chromosomes when donor DNA carrying homology regions flanking an endogenous *I-SceI* site was previously inserted in the mouse genome. Moreover, the microinjection of *I-SceI* together with a transgene flanked by meganuclease sites, increased the transgene integration efficiency in bovine embryos (Bevacqua et al., 2013). Lastly, a modified version of this meganuclease containing nuclear localization sequence (NLS) was successfully used to generate transgenic pigs by cytoplasmic injection (Wang et al., 2014).

The most recent developments have been the programmable endonucleases that resulted from the fusion between *Fok1* (Li et al., 1992), and DNA recognition domains such as the zinc finger (ZFN, Kim et al., 1996) and the transcription activator-like effector (TALEN, Christian et al., 2010). Initially, they were used as an efficient modification method to obtain edited somatic cells prior to SCNT (Hauschild et al., 2011; Carlson et al., 2012), and later on, both ZFN and TALEN were directly injected into the zygote as mRNA (Lillico et al., 2013; Tan et al., 2013) to induce specific genetic modifications allowing the expansion of knock-out pig models. However, before these efficient techniques could be spread throughout the scientific community, a much simpler technique was developed.

### Genetic Modification of Animals in the New AC (After CRISPR) Era

CRISPR-Cas systems were the most recently programmable endonuclease-based genetic engineering tools developed, practically monopolizing the gene editing field, since these new systems are more efficient, cheaper and simpler than the previous ones (Knott and Doudna, 2018). The year 2013 is considered to be the first year of a new era, the AC era.

Although the discovery of the CRISPR systems can be deemed to be serendipitous, because rare repeat sequences were observed by sequencing bacterial genes (Ishino et al., 1987; Mojica et al., 1993), the CRISPR-Cas systems were developed after a decade of combined efforts of many researchers who translated their knowledge into a revolutionary molecular biology tool, with a huge impact on many scientific fields (Mojica et al., 2005; Barrangou et al., 2007; Jinek et al., 2012; Cong et al., 2013; reviewed by Lander, 2016).

In almost all archaebacteria and half of bacteria, a huge diversity of CRISPR-Cas systems has been found, described and classified (Makarova et al., 2020). The CRISPR-Cas9 of *Streptococcus pyogenes* (SpCRISPR-Cas9) is one of the most used tool (Cong et al., 2013; reviewed by Marraffini, 2016), both in its original version with two RNAs, the CRISPR RNA and the transactivating CRISPR RNA (crRNA and tracrRNA, respectively) (Cong et al., 2013) or with just one RNA, known as single-guide RNA (sgRNA), a synthetic chimera between crRNA and tracrRNA (Jinek et al., 2012). In the CRISPR-Cas systems, where a single protein is used, target specificity is given by the sequence present in the crRNA or sgRNA (of 20 bp long); therefore, by simultaneously introducing different sgRNAs, several locus modifications are possible at the same time (Cong et al., 2013). Although other CRISPR-Cas systems have been described and used (Kotani et al., 2015), we will focus on SpCRISPR-Cas9, whose only genomic sequence requirement is the presence of an NGG sequence known as the protospacer adjacent motif (PAM), close to the cut site. Considering the CCN triplet in the antiparallel strand, and a random distribution of the four nucleotides, a PAM will be found every 8 nucleotides.

Moreover, following the completion of the Human Genome Project (Green et al., 2015) an accelerated development of cheaper and faster methods converted the Next Generation Sequence (NGS) techniques in standard tools for many applications in clinical and agronomical research (van Dijk et al., 2018). Along with the huge availability of sequences, there are a lot of *in silico* tools that allow the identification of homologous genes between species (Chen and Coppola, 2018).

The available sequence data embraced the development of many online programs that allow for the design of the most convenient guides to perform double-strand breaks at a specific locus, reducing the chances of off-target or undesired breaks (Cui et al., 2018). However, around 10% of the designed guides are not able to drive a precise DSB in mouse zygotes (Yuan and Hu, 2017). This can be explained by a more complex chromatin DNA structure in mammals than in bacteria or phages which are natural substrates for this nuclease. Therefore, the simple screening of guides is required (shown below).

Programmable endonuclease can also facilitate the insertion of exogenous sequences in a specific locus (Mali et al., 2013), producing transgenic animals; however, this strategy will not be discussed in this review.

## The Road to Obtain an Edited Pig in the New AC era

The easy application of the CRISPR-Cas editing tools promoted the generation of many animal models that were impossible to develop before, such as domestic animals and even, unfortunately, humans. However, the “*replacement*” principle, one of the 3Rs principles of animal welfare, does suggest looking for alternative approaches, such as the use of *in vitro* cell cultures or the generation of rodent models, to answer some biological questions.

Nevertheless, pigs are considered a great promise in biomedical research, since they are interesting models for human diseases and the best option as an organ supply for xenotransplantation. Thus, gene-edited pigs have become an effective and, in some cases, irreplaceable tool. In order to produce them it is necessary to complete the following three stages: (a) the design of efficient programmable nucleases, (b) the generation of edited single-cell embryos, and (c) the subsequent editing analysis of the piglets produced.

### Efficiency of the CRISPR-Cas9 System

As it has been already mentioned, the specificity of CRISPR-Cas9 depends on the crRNA or the sgRNA, and there are several publications describing how to synthesize them (Ran et al., 2013; Fujihara and Ikawa, 2014; Jacobi et al., 2017). In this section, different strategies to evaluate the efficiency of CRISPR-Cas9 will be discussed.

The simplest assays are based on the use of DNA plasmids, as a binary system, encoding for Cas9 and for the sgRNA, respectively (Mali et al., 2013). Mashiko et al. (2013) described a tool for quantifying the efficiency of CRISPR-Cas9 based on the reconstitution of *gfp* functionality after a DSB in episomal plasmid constructions. However, in order to mimic the real conditions, an analysis of editing efficiency should be conducted in the porcine genome, using cell lines or *in vitro-*produced embryos (see below in the Porcine zygote production section). Although there are few exceptions, the use of CRISPR-Cas9 plasmids is normally limited to somatic cell cultures (Wang K. et al., 2015). In embryos, due to the transcription arrest until the first mitotic cycles, the use of RNAs or the RNP (ribonucleoprotein) complex is preferred (Hai et al., 2014). In addition, CRISPR-Cas9 *in vitro* digestion can be used as a pre-validation of the system to induce a DSB in a target site. This assay is only applicable for RNP format, and consists of the *in vitro* assembly of the Cas9 protein with the *in vitro* transcribed or chemically synthetized sgRNA or crRNA: tracrRNA duplex, followed by the digestion reaction with the fragment that contains the target site (Mehravar et al., 2019). In cell culture assays, the selection marker commonly carried by the Cas9 coding plasmids can be used after transformation to enrich the culture for transformed cells (Zhou et al., 2015; Bevacqua et al., 2016; Yin et al., 2019). The analysis of these results, which tend to have a high background level, are complex because the obtained cells have different editing events. On the contrary, the *in vitro-*produced embryos have a small number of cells (around 50) derived from a few editing events. These results tend to be clearer and allow the study of features such as mosaicism or heterozygosity (Sakurai et al., 2014; Whitworth et al., 2014; Bevacqua et al., 2016). An animal or embryo is mosaic when not all of its cells have the same genotype, and this happens when gene editing occurs after the first embryonic mitotic divisions. In these cases, more than two alleles per locus can be detected.

In all these assays, the genotype characterization of the resulting cells initiates with an amplification of the edited locus through a PCR reaction. The primers should be designed so that they flank the target site. Since deletions produced in the process of DSB repair can involve hundreds of nucleotides, a primer design far enough from the target sites is recommended to ensure a correct hybridization, even within large deletion events. Optimal primers anneal at least 200 nt. away from the intended cutting sites (Mianné et al., 2017). Moreover, nested PCR is a good choice when the amount of DNA in the samples is limited. The second step is the analysis of the amplified DNA. Although the PCR amplicons could be screened directly by Sanger sequencing, some indirect strategies have been developed allowing massive and inexpensive tests. When the efficiency is low, these assays are an excellent alternative for sorting samples prior to Sanger sequencing.

The use of two sgRNA flanking an essential element in the targeted gene (dropout knock-out, Chen et al., 2014; Low et al., 2016) allows a simple evaluation of the designed sgRNA. In this case, the double cut induces an internal deletion that can be verified by a change in electrophoresis mobility of the new smaller resulting amplicon. However, these tests underestimate the rates of non-functional allele formation, because single or double cuts repaired with indels occur without the internal deletion, and therefore, these cases are indistinguishable from the wild type on an agarose gel electrophoresis.

Single cuts (indels) can also be analyzed by heteroduplex formation assays. These techniques distinguish between amplicons that carry mutations from those which do not. However, these methods do not provide information about the number or the composition of the alleles present. Heteroduplex formation assays consist in denaturing and annealing together wild-type and mutant amplicons (or amplicons that carry two different mutations), creating a bubble due to the mismatched chains. Heteroduplex DNAs can be analyzed by using nucleases such as T7 endonuclease 1 (T7E1) (Mashal et al., 1995) or Surveyor nuclease (an enzyme from the CEL nuclease family, Qiu et al., 2004). These nucleases recognize a mismatch site and, consequently, cleave both DNA strands. Then, the products of enzyme digestion are resolved by agarose gel electrophoresis showing a full-length amplicon (due to the presence of homoduplexes) and the expected-size cleavage products, if Cas9 cleavage occurred producing indels (Harms et al., 2014). Heteroduplex DNAs could also be analyzed by the heteroduplex mobility assay (HMA) (Ota et al., 2013, 2014). Since heteroduplexes have an open single-strand configuration surrounding the mismatched region, they can be separated from homoduplexes by polyacrylamide gel electrophoresis because of changes in complex migration patterns. In some cases, when an induced mutation is well represented in an allele pool, it is necessary to introduce a wild-type amplicon before heteroduplex formation to increase the accuracy of the method (Sentmanat et al., 2018). Another indel detection assay is the high-resolution melting analysis (HMRA) (Bassett et al., 2014). HMRA uses the different melting temperatures of the wild-type and a mutant amplicon to distinguish one from another, using a melting curve analysis with a fluorescent dye that fluoresces brightly when specifically bound to double-stranded DNA (Wittwer et al., 2003).

The Indel Detection by Amplicon Analysis (IDAA) is a sensitive and accurate technique that provides detailed information on cleavage efficiency, size and nature of the allelic variants generated (Yang et al., 2015). The technique is based on a single-step tri-primer PCR, where a universal 6-FAM 5-labeled primer (FamF) designed to target the forward primer in a specific extension is used. This technique results in the labeling of FAM amplicons that can be detected using standard DNA fragment analysis by the capillary electrophoresis methodology (Andersen et al., 2003).

In the case of defined nucleotide changes or specific point mutations, additional silent mutations, which do not alter the amino acid sequence of the encoded protein, can be included in the donor DNA to create new restriction sites. In this way, the amplified DNA at the target locus can be digested with the corresponding new restriction enzyme to detect point mutations by homology-directed repair (HDR) events (Wang et al., 2013). Nevertheless, imperfect or incomplete HDR events can occur, leading to undesired sequence modifications near the target site (Mianné et al., 2017). Similarly, if the chosen sgRNA cuts in a restriction enzyme site when the indels are generated, the restriction site could be lost.

Finally, the sequencing of the target regions of the alleles present in the sample is necessary to obtain a complete characterization. Chromatograms from direct Sanger sequencing of PCR products can be easily analyzed when samples contain only one or two possible alleles, such as clonal cell cultures and F1 animals. In samples that could contain more than two alleles (mosaicism), such as F0 animals, or polyclonal cell cultures, it is often difficult to determine the sequences of the alleles present. In this regard, different algorithms were developed to help in these analyses. The Tracking of Indels by Decomposition (TIDE) is an algorithm that analyzes. Sanger sequence traces, identifies the major induced mutations in a target site, and determines their frequency in a cell population (Brinkman et al., 2014; Ryczek et al., 2020). It is a simple, rapid and cost-effective method compared to sub-cloning individual amplicons of the target region and sequencing enough numbers of them to obtain an accurate characterization of the indel spectrum, which is more labor-intensive and expensive. A modified version of TIDE, the Tracking of Insertion, DEletions, and Recombination events (TIDER), estimates the frequency of targeted small nucleotide changes introduced by CRISPR in combination with HDR using a donor template (Brinkman et al., 2018).

### Generation of Single-Cell Edited Embryos

One of the first decisions to be made for the generation of pigs with specific gene modifications is whether to edit somatic cells to be used for cloning or directly introduce the CRISPR-Cas9 components into the zygotes. The advent of this new genome editing technology promotes the use of both strategies and the choice of one over the other will depend on the laboratory capacities.

#### SCNT

The development of pig cloning (Onishi et al., 2000) opened the possibility of generating homogeneous animals with modifications incorporated into somatic cells (Lai et al., 2002). In addition, in order not to depend on specific equipment and to be able to increase the number of reconstituted embryos, the handmade cloning (HMC) technique has been useful (Vajta et al., 2001; Du et al., 2007). The main distinctive feature of HMC is the use of sharp blades for bisection of zona-free oocytes under stereomicroscope instead of using a micromanipulator to enucleate them.

In either methodology, traditional cloning (TC) and HMC, the results obtained still show a low efficiency to produce cloned piglets (with only 0.3–2% of transferred embryos developing to term; Du et al., 2007; Zhang et al., 2012; Liu T. et al., 2015; Gadea et al., 2020). In this regard, the aggregation of three zona-free reconstructed cloned embryos was proposed as a strategy to improve embryo development, quality (Buemo et al., 2016) and deliveries (Siriboon et al., 2014) in TC and HMC, respectively. Despite the limitation of both techniques, they are used to generate edited pigs with CRISPR/Cas9. Somatic cells, such as fetal fibroblasts, are transformed with plasmids encoding for the Cas9 and the sgRNAs, along with a reporter gene and/or an antibiotic resistance gene; allowing the screening and/or selection of the modified cells (Ren et al., 2019). Once the edited cells are obtained, they are used to generate founder pigs, which will present a predictable genotype avoiding mosaicism (Chen et al., 2015; Wang K. et al., 2015; Kumbha et al., 2020). Furthermore, the multi-targeting capacity of the CRISPR-Cas9 system allows to edit many target genes simultaneously, a feature used by Niu et al. (2017), to produce porcine retrovirus PERV-free pigs by SCNT, where 62 copies of this retrovirus were edited.

Another interesting alternative is to retrieve fetuses generated by CRISPR-Cas9 delivery into porcine zygotes and screen the fetal fibroblasts for the specific modifications. These selected cells will then be used for performing SCNT carrying the desired modifications, avoiding mosaic animal generation and the laborious enrichment and selection process of edited cells from primary cultures (Kang et al., 2016).

#### Porcine Zygote Production

The new genetic editing tools are now so efficient that allow zygotes direct modification. For this reason, besides cloning, other embryo production techniques, such as IVF or *in vivo* zygote retrieval, are promoted as good alternatives for the generation of genetically modified pigs. The different methodologies to obtain the porcine embryos, as well as the delivery options to introduce the CRISPR-Cas9 system into them, will be further discussed in this section.

##### Production of parthenogenetic embryos

Parthenogenetic activation is an alternative to *in vitro* embryo production since embryos are capable of developing to blastocysts, like fertilized oocytes (Kure-Bayashi et al., 1996), avoiding variations due to the sperm factor (Gupta et al., 2008). These embryos have been proposed to evaluate *in vitro* the efficiency of gene editing tools (Tao et al., 2016), although these embryos are not viable to generate offspring.

Oocyte activation can be artificially induced by simulating the effects produced by the sperm. The protocols commonly used for this procedure are based on the exposure of oocytes to agents that promote the increase in cytoplasmic levels of Ca 2 +. Following exposure to Ca 2 + inducing agents, oocytes are often treated with inhibitors of protein synthesis (e.g., cycloheximide – CHX) or kinase activity (e.g., 6-dimethylaminopurine – 6-DMAP) generating a diploid parthenogenetic embryo that will be able to develop to the blastocyst stage (Alberio et al., 2001).

Electrical stimulation is commonly used to activate pig oocytes and, in order to optimize this method, the combination of electrical and chemical activation protocols have been proposed to produce transgenic embryos (More details of these protocols are described in Liu S. et al., 2015).

Another important application of parthenogenetic embryos is as a supplementary source to improve maternal recognition, pregnancy and implantation rates of SCNT in pigs (De Sousa et al., 2002; Kawarasaki et al., 2009).

##### *In vitro* fertilization (IVF)

Despite the enormous effort and progress, the current *in vitro* fertilization system remains inefficient giving as a result low embryo development and low-quality blastocysts compared to the *in vitro* systems from other species such as bovine or mouse (reviewed by Gil et al., 2010; Grupen, 2014). This is mainly due to the high incidence of polyspermy that occurs during IVF. Over the last 2 decades, many groups have been working to find a methodology to improve IVF and reduce polyspermy (reviewed by Funahashi, 2003; Romar et al., 2016). More recently, Li et al. (2018) showed that by simply reducing sperm concentration in the presence of cumulus cell, an improvement in fertilization (monospermy rate and normal pronuclear formation) and blastocyst formation were obtained. Moreover, IVF systems based on some *in vivo* conditions, such as a higher pH, and the presence of oviductal and follicular fluid and cumulus cell secretions, reduce polyspermy and increase the final embryo production (Soriano-Úbeda et al., 2017). A reason for this improvement may be due to the presence of extracellular vesicles in the porcine oviductal fluid (Alcântara-Neto et al., 2020). Nevertheless, several gene-editing studies use *in vitro* derived embryos, since they are less costly and time-consuming, and a large number of oocytes can be recovered from slaughterhouse ovaries. Considering the incidence of polyspermia, a method to isolate monospermic zygotes to avoid editing and transfer of polyspermic embryos is very useful. This can be achieved by identifying normal pronuclear formation by visualization in presumptive zygotes. A problem is that porcine zygotes exhibit a large amount of cytoplasmic lipid droplets. Therefore, zygote centrifugation after IVF was proposed as a simple non-invasive method to visualize pronuclei to identify two and poly-pronuclear zygotes (Wall et al., 1985). This technique allowed Gil et al. (2013) to identify 2 pronuclear zygotes, and to improve blastocyst quality and pregnancy efficiencies (number of live piglets per total transferred embryos) when these embryos were transferred to recipient gilts in comparison to non-centrifuged, non-selected zygotes in the control group.

##### *In vivo* zygote production

It is known that the development of *in vitro* pre-implantable mammalian embryos is compromised compared to those produced *in vivo*, presenting a delay in blastocyst development and fewer cells in the embryos (Macháty et al., 1998; Holm et al., 2002). Unfortunately, the available data on the effectiveness of *in vivo-*derived porcine zygote collection procedures remain limited to date. In this regard, some key aspects to take into account are the formation of pronuclei, which occurs between 3 and 5 h after fertilization and the first mitotic division that occurs 14–16 h later (Hunter, 1974). Therefore, the window for the collection of zygotes to be edited turns out to be very narrow. To perform this procedure, it is necessary to previously synchronize the estrus and ovulation of multiparous sows. Weaning is an effective physiological method, obtaining a fertile estrus between 3 and 5 days after weaning. To increase the number of fertilized oocytes, superovulation can be induced with equine chorionic gonadotropin (eCG) 24 h after weaning followed by human chorionic gonadotropin (hCG) administration. Then, females are submitted to post cervical insemination twice, at 6 and 24 h after the onset of estrus. For zygotes collection, sows are submitted to a surgical procedure in which they are anesthetized, their genital tracts are exposed through mid-ventral laparotomy, and zygotes are finally retrieved by flushing each oviduct. In this regard, Martinez et al. (2020) managed to recover a range between 69.0 and 73.3% of zygotes. However, the above-described procedures involve the need for specialized technicians and veterinarians and adapted facilities with sterile operating rooms, which for some groups could mean a budgetary limitation.

#### CRISPR-Cas Delivery Methods in Zygotes

Initially, the traditional procedures to deliver the editing tools into the zygotes involved microinjection. Several scientists have tried to develop newer, simpler and cheaper methods and some of these developments have been partially successful. A recent approach includes an electroporation-based method that bypasses microinjection with promising results obtained by numerous groups. In either case, the ultimate goal is to produce biallelic and homogeneous edited animals and, for this reason, timing for CRISPR-Cas9 system action, relative to DNA replication in the zygote, may be the most relevant event to be taken into account to reduce or eliminate mosaicism. The most commonly used methods will be compared in this section.

##### Intracytoplasmic microinjection

This technique is the most widely used for the generation of different animal models through the years. It consists in the microinjection of editing tools into presumptive one-cell stage embryos produced *in vivo* or by IVF. This technique requires the use of expensive micromanipulation equipment and skilled personnel to operate it. Additionally, it is time-consuming, the reason why the number of zygotes microinjected per repetition will be limited. As has already been mentioned, microinjection of mRNA for CRISPR-Cas9 or RNP is preferable to edit porcine zygotes (Sato et al., 2015; Jacobi et al., 2017; Lamas-Toranzo et al., 2019; Tanihara et al., 2019b). However, plasmids encoding for Cas9 nuclease and for sgRNA have also been used for this purpose (Petersen et al., 2016). The main problem of using plasmid DNA is that it lasts longer inside the cells, potentially increasing off-target mutations.

Considering the IVF limitations already described, some groups prefer to directly collect and microinject CRISPR-Cas9 tools into *in vivo-*produced presumptive zygotes close to insemination; and transfer the embryos into the oviduct immediately after microinjection to improve viability and pregnancy rates very good results (Hai et al., 2014; Wang Y. et al., 2015; Yu et al., 2016).

The main advantage of combining *in vitro-*produced embryos with microinjection as delivery technique of choice, is the possibility to exploit the narrow time window between gamete fusion and first embryo cell division to deliver editing tools. Thus, in order to reduce mosaicism without affecting embryo viability, several studies have been performed to evaluate the best timing to introduce the CRISPR-Cas9 system throughout the *in vitro* embryo production procedure. Tanihara et al. (2019b) concluded that the optimal moment to microinject CRISPR-Cas9 components as the RNP complex into zygotes was 6 h after the start of IVF, when the highest mutation rates were obtained without compromising embryo viability. Furthermore, a higher RNP complex concentration was shown to increase efficiency and biallelic mutations (although still low: 16.7%) in the resulting blastocysts (Tanihara et al., 2019b). Another group reached similar conclusions working with parthenogenetically activated oocytes. They observed that the best moment to microinject the CRISPR-Cas9 components as RNA was 6 h after activation, regarding blastocyst and mutation rates. However, no improvement in mosaicism was observed in this case (Sato et al., 2018). In contrast, Tao et al. (2016) showed a much significant improvement in the rates of biallelic mutation (93%) in embryos when CRISPR-Cas9 mRNA was microinjected 8 h after parthenogenetic activation.

In addition, in a recent study by Su et al. (2019), a microinjection of the CRISPR-Cas9 components as RNA into germinal vesicle porcine oocytes was proposed as a solution to reduce mosaicism. These oocytes were then *in vitro* maturated and parthenogenetically activated or fertilized by IVF. By applying this strategy, up to 83% of the mutant embryos obtained were non-mosaic, having no detrimental effect on embryo viability. Another particular approach is the injection of CRISPR-Cas9 system in reconstituted presumptive zygote (Sheets et al., 2016). In this case, without any selection, 6 out of 6 piglets carried a biallelic modifications.

Although this technique is widely applied for the generation of edited animal models, it requires the use of expensive micromanipulation equipment and skilled personnel to operate it. Additionally, it is time-consuming, the reason why the number of zygotes microinjected per repetition will be limited.

##### Embryo electroporation

More recently, this technique was developed for embryos, and it has grown in importance, proving to be cheaper and simpler than embryo microinjection for introducing indel mutations, large deletions, and small insertions (Kaneko and Mashimo, 2015; Kaneko, 2018). Recent studies have demonstrated that zona pellucida weakening is not necessary to achieve porcine zygotes gene editing by electroporation; preserving the integrity and viability of the embryo. There are mainly two different electroporators that have yielded good results, the CUY21EDIT II electroporator (BEX) (Nishio et al., 2018; Tanihara et al., 2019a,c) and the NEPA21 electroporator. The latter proposes to reduce the damage to embryos by using a three-step electrical pulse system. The first pulse, the poring pulse, makes micro-holes in the zona pellucida and oolemma of the embryos. The second pulse, the transfer pulse, transfers the endonucleases into the cytoplasm of the embryos. The third pulse, the polarity-changed transfer pulse, increases the opportunity of introducing the endonucleases into the embryos (Kaneko, 2017).

Although this technique is yet to generate sufficient data, it has shown good results allowing a faster gene editing of a bigger number of oocytes or zygotes at the same time, in contrast to the IP microinjection. The combination of a massive embryo production by IVF with the GEEP (gene editing by electroporation of Cas9 protein) technique compensates the poor IVF results with the fast editing rate by electroporation. This permits to transfer up to 200 embryos per recipient, finally obtaining living offspring with the intended gene target modifications (Tanihara et al., 2019a,c).

In addition, the success of this technique is in part due to its combination with Cas9 as protein, since the compact nature of the RNP complex seems to easily enter through the pores generated in zygotes in contrast to large Cas9 mRNA or other editing tools.

### Piglet Gene Editing Analysis

Except for edited animals by SCNT, where mosaicism is not an issue, the analysis of F0 is not a simple task (Teboul et al., 2017; Mehravar et al., 2019). It is very likely that F0 individuals could be mosaics; therefore, theoretically, the result of whether they are edited or not may depend on the tissue analyzed. Mosaicism in F0 animals could be responsible for differences between biopsied tissue and its germline; thus, producing F1 offspring without the expected genotype. The most obvious negative consequence will be a non-edited progeny after breeding. One of the first works to study mosaicism was carried out in mice, taking advantage of the *Tyr* gene whose loss of function generates albino phenotypes (Yen et al., 2014). Using CRISPR-Cas 9 in Tyr^±^ heterozygous zygotes with a mutation in a different exon, 6/12 pups were albinos (50%), 4/12 were pigmentation mosaics (33%), and 2/12 were fully pigmented (∼17%), and by analyzing DNA tail biopsies, more than 2 different alleles (up to 5) were found, even in homogenous animals (Yen et al., 2014). The backcrosses with homozygous albinos gave F1 homogeneous albino animals for all three mosaic phenotype animals, and unexpectedly for one of the phenotypically homogeneous colored animals too (Yen et al., 2014).

For this reason, in mice, there are authors who suggest analyzing, due to genotypic mosaicism, both the tail and the germline to track down false positives or negatives and to save time and money (Oliver et al., 2015). However, the risk of affecting the reproduction of these animals has slowed down the biopsies of gonads, especially in females. As an alternative to gonadal biopsies, performing ear biopsies is suggested, combined with a TIDE analysis of their sequences (Brinkman et al., 2014), or with deep sequencing by generating a DNA sequencing library with labeled primers to perform thousands of reads for each locus (Yen et al., 2014; Wei et al., 2018).

A good characterization of the founder animals allows to save money and time, and it is important in order to facilitate the decision of which animals to cross to obtain the correct F1. Breeding two F0 edited individuals can reduce the time to obtain a homozygous and homogeneous animal; nevertheless, the analysis can be more complex. In spite of the characterization, genotyping all F1 animals is recommended for the expected modification through Sanger sequencing of the targeted loci (Mianné et al., 2017).

## Concluding Remarks and Future Perspectives

**FIGURE 1:**
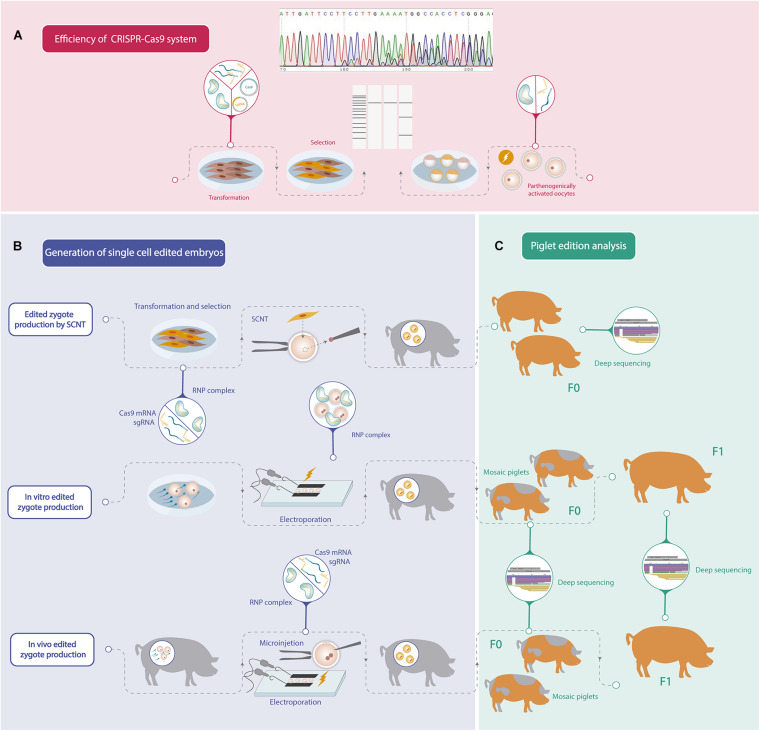
Schematic workflow of the different steps needed to generate a gene edited pig. **(A)** Efficiency analysis of mutations induced by CRISPR-Cas9 system. **(B)** Different strategies to generate one-cell stage porcine edited embryos. **(C)** Gene editing analysis of the founder pigs (F0) and offspring produced by crossbreeding F0 pigs (F1).

Recent advances in genome editing technology have accelerated the production of genetic modified pigs for different purposes by using several strategies. Although remarkable progress has been achieved in porcine gene editing, further improvements could still be achieved in order to increase biallelic mutation efficiency. In addition, since porcine reproduction is highly efficient, the application of assisted reproductive technologies has not been developed enough, and consequently protocols for oocyte *in vitro* maturation, IVF or embryo culture can still be improved. For these reasons, porcine gene editing strategies continue to be challenging, and each group should find its own road to generate an edited pig considering their strengths. The steps to follow in order to obtain an edited pig are summarized in Figure 1.

Cloning allows to obtain homogeneous animals with biallelic modifications; however, the birth rates of cloned piglets are still low. In addition, the successful generation of porcine expanded potential stem cells opens up new possibilities to simplify future strategies for the generation of edited pigs.

Direct zygote gene editing is a widely used approach because of the higher rates of healthy piglets, although some of them are mosaic. Another alternative is gene editing of *in vitro-*produced embryos by IVF, in combination with electroporation to deliver CRISPR-Cas9 components that seem to be a good and simple strategy, allowing to work with a larger number of embryos that compensate for the poorer development rates of these zygotes. A promising alternative is to obtain *in vivo* zygotes, which exhibit higher viability than *in vitro* embryos, followed by electroporation or microinjection of CRISPR-Cas9 components to ensure higher rates of viable edited embryos. However, this procedure involves additional costs related to the donor animals.

Recently, as a future perspective, some modifications of the CRISPR-Cas9 system are emerging (reviewed by Anzalone et al., 2020). A new chimera Cas9 protein that is capable of editing nucleotide conversions without DSB, has also been used to edit pigs (Xie et al., 2019). Moreover, epigenetic modifications are now possible by using dCas9 (Xu et al., 2020) that have been proposed to improve the viability of cattle embryos *in vitro* (Savy et al., 2020) and could be an effective tool to apply in porcine embryo production. Finally, by improving ICSI technique in pigs, ICSI mediated-gene editing would be an interesting option for the generation of edited piglets since it was demonstrated that when the delivery of the CRISPR-Cas9 system was done during ICSI in humans, mosaicism was reduced in the resulting embryos (Ma et al., 2017).

Nowadays, the simplicity of the new editing tools allowed the democratization of their use for the generation of edited pigs in laboratories around the world. These gene-edited animals cannot be differentiated from spontaneous mutants, since no exogenous genes are introduced and they should not be regulated at all or their regulation should be less strict than for transgenic animals (Van Eenennaam et al., 2019). However, few national regulatory agencies distinguish between genetically modified organisms and edited organisms. This distinction could greatly impact on the edited animal research, especially for agricultural purposes.

## Author Contributions

LR and RF-M took part in manuscript writing and editing. DS took part in the manuscript edition. All authors took part in the conceptualization of the review.

## Conflict of Interest

The authors declare that the research was conducted in the absence of any commercial or financial relationships that could be construed as a potential conflict of interest.
